# BeetRepeats: reference sequences for genome and polymorphism annotation in sugar beet and wild relatives

**DOI:** 10.1186/s13104-024-06993-4

**Published:** 2024-11-27

**Authors:** Nicola Schmidt, Sophie Maiwald, Ludwig Mann, Beatrice Weber, Kathrin M. Seibt, Sarah Breitenbach, Susan Liedtke, Gerhard Menzel, Bernd Weisshaar, Daniela Holtgräwe, Tony Heitkam

**Affiliations:** 1 Faculty of Biology, Technische Universität Dresden, 01069 Dresden, Germany; 2 Fakulty of Biology & CeBiTec, Universität Bielefeld, 33615 Bielefeld, Germany; 3 Institute of Biology I, RWTH Aachen University, 52056 Aachen, Germany

**Keywords:** Sugar beet, *Beta vulgaris*, *Patellifolia*, Repetitive DNA, Transposable elements, Satellite DNAs, Genome annotation

## Abstract

**Objectives:**

Despite the advances in genomics, repetitive DNAs (repeats) are still difficult to sequence, assemble, and identify. This is due to their high abundance and diversity, with many repeat families being unique to the organisms in which they were described. In sugar beet, repeats make up a significant portion of the genome (at least 53%), with many repeats being restricted to the beet genera, *Beta* and *Patellifolia*. Over the course of over 30 years and many repeat-based studies, over a thousand reference repeat sequences for beet genomes have been identified and many experimentally characterized (e.g. physically located on the chromosomes). Here, we present the collection of these reference repeat sequences for beets.

**Data description:**

The BeetRepeats_v1.0 resource is a comprehensive compilation of all characterized repeat families, including satellite DNAs, ribosomal DNAs, transposable elements and endogenous viruses. The genomes covered are those of sugar beet and closely related wild beets (genera *Beta* and *Patellifolia*) as well as *Chenopodium quinoa* and *Spinacia oleracea* (all belonging to the Amaranthaceae). The reference sequences are in fasta format and comprise well-characterized repeats from both repeat categories (dispersed/mobile as well as tandemly arranged). The database is suitable for the RepeatMasker and RepeatExplorer2 pipelines and can be used directly for any repeat annotation and repeat polymorphism detection purposes.

**Supplementary Information:**

The online version contains supplementary material available at 10.1186/s13104-024-06993-4.

## Objective

Due to its roles in beet evolution and variability, sugar beet’s (*Beta vulgaris* subsp. *vulgaris*) repeatome has been a subject of interest for over 30 years. Starting with the detection of repeat-derived ladder patterns using Southern hybridization experiments [1], more detailed studies became possible, including wet lab (i.e. fluorescent in situ hybridization; e.g. [2, 3]) as well as bioinformatics methods (i.e. read clustering [4–6]).

A significant portion (at least 53% [5]) of the sugar beet genome consists of repeats, comprising a great number of different transposable elements (TEs) as well as tandem repeats. Compiling and unifying data from over 50 publications and theses, we here provide a downloadable and easy-to-use resource of the repeat profiles in beet genomes [7].

With this data note, we provide a comprehensive collection of all characterized repeat families in sugar beet and closely related wild beet genomes [7], facilitating genome annotation as well as the investigation of evolutionary trajectories of TEs and their host genomes.

## Data description

We have collected repetitive DNA sequences that are representative for all major repeat families in genomes of the crop sugar beet (*Beta vulgaris* subsp. *vulgaris*) and related wild beets (genera *Beta* and *Patellifolia*), and provide them in fasta format [7]. Furthermore, we added repeats identified in two further Amaranthaceae species (*Chenopodium quinoa* and *Spinacia oleracea*). In detail, the beet repeatomes are represented by:

- 223 non-long terminal repeat (non-LTR) retrotransposons, with 100 long interspersed nuclear elements (Belline LINEs) [8, 9] and 123 short interspersed nuclear elements (AmaSINEs) [10];
- 61 non-autonomous LTR retrotransposons, with 60 terminal repeat retrotransposons in miniature (TRIMs) [7, 11] and one large retrotransposon derivate (LARD) [7];
- 355 Ty1-*copia* retrotransposons, with 220 Retrofit sequences, 11 Oryco/Ivana sequences, 87 Tork sequences, 9 SIRE sequences, and 28 Bianca sequences [7, 12, 13];
- 69 Ty3-*gypsy* retrotransposons, with 25 chromoviruses [3, 14], 27 errantiviruses/Athila [15], and 17 Tat sequences [7];
- 3 endogenous pararetroviruses (beetEPRVs) [16];
- 299 DNA transposons, with 12 EnSpm/CACTA sequences [17], 3 autonomous and 116 non-autonomous hAT sequences (BvhAT and BvhATpin MITEs) [18], 51 autonomous and 90 non-autonomous PIF/Harbinger sequences (BvPIF/Pong and BvPIF/Pong MITEs) [7], one autonomous and 24 non-autonomous Tc1_Mariner sequences (Vulmar and VulMITEs) [17, 19], and 2 helitron sequences [7];
- 82 satellite DNAs [2, 5, 20–31];
- 21 minisatellites/tandem repeats [7, 27, 32, 33];
- 3 rDNAs (two variants of the 5S rDNA and one 45S rDNA sequence) [34, 35].

This list is further detailed in Data file 1 (see Table 1).

**Table 1:** Overview of data files/data sets

Label	Name of data file/data set	File type (file extension)	Data repository and identifier (DOI or accession number)
*Data set 1*	BeetRepeatDB_v1.0.fasta	*Fasta file (.fa)*	Zenodo (https://doi.org/10.5281/zenodo.8255813) [7]
*Data set 2*	BeetRepeatDB_v1.0_at_EL10.gff	*GFF file (.gff)*	Zenodo (https://doi.org/10.5281/zenodo.8255813) [7]
*Data set 3*	BeetRepeatDB_v1.0_at_2320BvONT_v1.0.gff	*GFF file (.gff)*	Zenodo (https://doi.org/10.5281/zenodo.8255813) [7]
*Data set 4*	BeetRepeatDB_v1.0_at_RefBeet1.5.gff	*GFF file (.gff)*	Zenodo (https://doi.org/10.5281/zenodo.8255813) [7]
*Data file 1*	BeetRepeatDB_v1.0-Content.docx	*Microsoft Word Document (.docx)*	Zenodo (https://doi.org/10.5281/zenodo.8255813) [7]

The BeetRepeats fasta resource contains in silico consensus sequences as well as exemplary, representative copies. It is formatted to meet the requirements for a ‘custom repeat database’ utilized by the RepeatExplorer2 pipeline [36]. Due to the absence of a respective category in the RepeatExplorer2 annotation, all tandem repeats listed in our resource (except rDNA) were classified as satellite DNAs [7].

As there are several assemblies of the sugar beet genome available, we provide an annotation of the repeats from our database within the three different sugar beet assemblies EL10 [37], 2320BvONT_v1.0 [38], and RefBeet1.5 (https://jbrowse.cebitec.uni-bielefeld.de/RefBeet1.5/) as GFF files (see Table 1). To create these annotation files, we used the RepeatMasker pipeline [39] with standard parameters (performing softmasking instead of hardmasking and deactivating the low complexity masking).

## Limitations

- Whereas the repeatome of sugar beet should be completely covered with this database, repeat identification and characterization in wild beet genomes are still ongoing. Thus, the database is under constant expansion and requires regular updates.
- Due to different requirements for custom databases, it may be necessary to reformat our database in order to use it with certain software programs. For instance, to acquire a detailed summary table by RepeatMasker, additional information on repeat classification in the sequence names have to be shortened to only indicate the class and subclass of the respective repeat.
- Diverged repeated sequences (e.g. old and/or lowly abundant variants with an accumulation of mutations) are not completely covered by our representative sequences. Therefore, it is to be expected that repeat masking of beet genomes using our database results in lower repetitive genome proportions than the actual repeat fraction.
- Since we focused largely on beets, we included only few selected repeats from *C.* *quinoa* and *S.* *oleracea* in our resource [7]. The repeats from these more distantly related plants were only included, if they represented derivatives of the listed beet repeats.

## Supplementary Information

Additional file 1.

## Data Availability

The data described in this Data note can be freely and openly accessed on Zenodo under https://doi.org/10.5281/zenodo.8255813 [7]. Please see Table 1 for details and links to the data.

## Abbreviations

TE: Transposable element
LTR: Long terminal repeat
LINE: Long interspersed nuclear element
SINE: Short interspersed nuclear element
TRIM: Terminal repeat retrotransposon in miniature
LARD: Large retrotransposon derivate
EPRV: Endogenous pararetrovirus
MITE: Miniature inverted-repeat transposable element
rDNA: Ribosomal DNA
