# Supplementary material for: BeetRepeats: reference sequences for genome and polymorphism annotation in sugar beet and wild relatives
Source: BMC Res Notes. 2024 Nov 27;17:351. doi: 10.1186/s13104-024-06993-4 (PMC11603912; doi:10.1186/s13104-024-06993-4)
Supplement: Supplementary file 1 — Additional file 1. [file 13104_2024_6993_MOESM1_ESM.docx]

# BeetRepeatDB_v1.0

Content

- **SINEs:**123 sequences from *Beta* and *Patellifolia* genomes, incl. SINEs from *Chenopodium quinoa*
   and *Spinacia oleracea* (Schwichtenberg *et al.*, 2016)
- **LINEs:**100 Belline LINEs from *B. vulgaris* (Heitkam and Schmidt, 2009; Heitkam *et al.*, 2014)
- **non-autonomous LTR retrotransposons:**2 Ama Cassandra TRIMs from *B. vulgaris* (Maiwald *et al.*, 2020)
  58 further TRIMs from *B. vulgaris* (this database)
  1 Coro LARD from *B. corolliflora* (this database)
- **Ty1-*copia* retrotransposons:**220 **Retrofit** sequences:
   218 Retrofit sequences from *B. vulgaris* (this database)
   1 Tbv4 from *B. vulgaris* (Brandes *et al.*, 1997)
   1 Paco from *P. patellaris* (this database)
  11 **Oryco/Ivana** sequences from *B. vulgaris* (this database)
  87 **Tork** sequences
   84 Tork sequences from *B. vulgaris* (this database)
   1 SALIRE from *B. vulgaris* (Weber *et al.*, 2010)
   1 Gritchy from *B. vulgaris* (this database)
   1 Patty from *P. patellaris* (this database)
  9 **SIRE** sequences from *B. vulgaris* (this database)
  28 **Bianca** sequences from *B. vulgaris* (this database)
- **Ty3-*gypsy* retrotransposons:**25 **chromoviruses** from *B. vulgaris* (Weber and Schmidt, 2009; Weber *et al.*, 2013):
   13 CRM sequences (*Beetle*)
   3 Tekay sequences (*Bongo*)
   8 Reina sequences (*Bingo*)
   1 Galadriel (*Beon*)
  27 **errantiviruses/Athila** sequences from *B. vulgaris* (Elbe: Wollrab *et al.*, 2012)
  17 **Tat** sequences from *B. vulgaris* (this database):
   17 Ogre sequences (4 Fiona sequences, 13 Hagrid sequences)
- **endogenous pararetroviruses**4 beetEPRV sequences representing 3 different Florendoviruses from *B. vulgaris* (Schmidt *et al.*, 2021)
- **DNA transposons:**12 **EnSpm/CACTA** sequences from *B. vulgaris* (Jacobs *et al.*, 2004)
  119 **hAT** sequences from *B. vulgaris* (Menzel *et al.*, 2012):
   3 autonomous BvhAT sequences
   116 non-autonomous BvhATpin MITEs
  141 **PIF/Harbinger** sequences from *B. vulgaris* (this database):
   35 autonomous BvPIF sequences
   48 non-autonomous BvmPIF MITEs
   16 autonomous BvPong sequences
   42 non-autonomous BvmPong MITEs
  25 **Tc1_Mariner** sequences from *B. vulgaris*:
   1 autonomous Vulmar1 (Jacobs *et al.*, 2004)
   24 non-autonomous VulMITEs (Menzel *et al.*, 2006)
  2 **helitron** sequences from *B. vulgaris* (HEL: this database)
- **satellite DNAs:**7 beetSat01-pBV sequences from sect. *Beta* genomes
   (Zakrzewski *et al.*, 2011, Zakrzewski *et al.*, 2013; pBV-VII: this database)
  3 beetSat02-pEV sequences from *Beta* and *Patellifolia* genomes (Schmidt *et al.*, 1991;
   beetSat02-pEV_Corollinae: Schmidt *et al.*, 2023.; pAp11: Dechyeva *et al.*, 2003)
  1 beetSat03-pTS5 from *P. procumbens* (Schmidt and Heslop-Harrison, 1996)
  1 beetSat04-pTS4.1 from *P. procumbens* (Schmidt and Heslop-Harrison, 1996)
  1 beetSat05 from *Patellifolia* sp. (Schmidt *et al.*, 2023)
  25 beetSat06-pAv34 sequences from *Beta* and *Patellifolia* genomes
   (Dechyeva and Schmidt, 2006)
  3 beetSat07-pHC8 sequences (from *B. corolliflora*: Gindullis *et al.*, 2001;
   from *B. vulgaris* and *B. nana*: this database)
  1 beetSat08-BlSat01 from *B. lomatogona* (Ha, 2018)
  4 beetSat09-pHT sequences from *B. trigyna* (Schmidt and Heslop-Harrison, 1993)
  9 beetSat10-pRN sequences
   (from *B. nana*: Kubis *et al.*, 1997; from *B. vulgaris* and *B. corolliflora*: this database)
  1 beetSat11-BlSat05 from *B. lomatogona* (Ha, 2018)
  1 beetSat12-pBC1416 from *B. corolliflora* (Gao *et al.*, 2000)
  3 beetSat13 sequences (pBC1447 from *B. corolliflora*: Gao *et al.*, 2000;
   ChenSat01 from *C. quinoa*: Heitkam *et al.*, 2020)
  2 beetSat14 sequences from *B. corolliflora* (Gao *et al.*, 2000)
  1 beetSat15 from *Corollinae* sp. (Schmidt *et al.*, 2023)
  1 beetSat16 from *Corollinae* sp. (Schmidt *et al.*, 2023)
  2 beetSat17 sequences from *Corollinae* sp. (Schmidt *et al.*, 2023;
   BnSat01: this database)
  2 beetSat18 sequences from *Corollinae* sp. (Schmidt *et al.*, 2023;
   BnSat03: this database)
  1 beetSat19-Tantalos from *B. vulgaris* (Zakrzewski *et al.*, 2010, Zakrzewski *et al.*, 2014)
  13 further satellite DNAs:
   1 pHT49 from *B. trigyna* (Schmidt and Heslop-Harrison, 1993)
   4 different BlSat sequences from *B. lomatogona* (Ha, 2018)
   4 different BnSat sequences from *B. nana* (this database)
   4 ChenSat-02 sequences from *C. quinoa* (Heitkam *et al.*, 2020)
  21 minisatellites or tandem repeats:
   1 Dione from *B. vulgaris* (Zakrzewski *et al.*, 2010, Zakrzewski *et al.*, 2014)
   1 Niobe from *B. vulgaris* (Zakrzewski *et al.*, 2010, Zakrzewski *et al.*, 2014)
   4 different BvuSat sequences (Li *et al.*, 2021)
   15 different BvMSat sequences (BvMSat01, 03-05, 07-11: Zakrzewski *et al.*,
   2010; BvMSat02, 06, 12-15: this database)
   1 pBV tandem repeat from *B. corolliflora* (this database)
- **rDNA:**2 **5S rDNA** sequences from *B. vulgaris* (pXV: Schmidt *et al.*, 1994)
  1 **45S rDNA** from *B. vulgaris* (pZR18S: Paesold *et al.*, 2012)

Notes

Due to the absence of a respective category in the RepeatExplorer2 annotation (Novák *et al.*, 2020), all tandem repeats listed in the resource (except rDNA) were alternatively classified as satellite DNAs. Also, the column ‘Similarity hits to custom database’ created during RepeatExplorer2 analyses only lists the information following the hashtag in the sequence header. For instance: the LTR retrotransposon ‘Bongo1_ Beta_vulgaris#Ty3_gypsy/chromovirus/Tekay’ is only indicated as ‘Ty3_gypsy/chromovirus/Tekay’. More detailed information about the precise element and the belonging plant genome as indicated in the sequence headers can be found in the downloaded RepeatExplorer2 archive in the directory: "seqclust\clustering\clusters\dir_CLxxxx\custom_db_extra _database_annotation .csv".

References

Brandes A, Heslop-Harrison JS, Kamm A, Kubis S, Doudrick RL, Schmidt T. Comparative analysis of the chromosomal and genomic organization of Ty1-*copia*-like retrotransposons in pteridophytes, gymnosperms and angiosperms. Plant Mol Biol. 1997;33:11–21.

Dechyeva D, Gindullis F, Schmidt T. Divergence of satellite DNA and interspersion of dispersed repeats in the genome of the wild beet *Beta procumbens*. Chromosome Res. 2003;11:3–21.

Dechyeva D, Schmidt T. Molecular organization of terminal repetitive DNA in *Beta* species. Chromosome Res. 2006;14:881–897.

Gao D, Schmidt T, Jung C. Molecular characterization and chromosomal distribution of species-specific repetitive DNA sequences from *Beta corolliflora*, a wild relative of sugar beet. Genome. 2000;43:1073–1080.

Gindullis F, Desel C, Galasso I, Schmidt T. The large-scale organization of the centromeric region in *Beta* species. Genome Res. 2001;11:253–265.

Ha BH. Structure, organization, and evolution of satellite DNAs in species of the genera *Beta* and *Patellifolia*. Doctoral dissertation, Technische Universität Dresden. 2018. <https://nbn-resolving.org/urn:nbn:de:bsz:14-qucosa-238083>.

Heitkam T, Schmidt, T. BNR - a LINE family from *Beta vulgaris* contains an RRM domain in open reading frame 1 and defines a L1 subclade present in diverse plant genomes. Plant J. 2009;59:872–882.

Heitkam T, Holtgräwe D, Dohm JC, Minoche AE, Himmelbauer H, Weisshaar B, et al. Profiling of extensively diversified plant LINEs reveals distinct plant-specific subclades. Plant J. 2014;79:385–397.

Heitkam T, Weber B, Walter I, Liedtke S, Ost C, Schmidt T. Satellite DNA landscapes after allotetraploidization of quinoa (*Chenopodium quinoa*) reveal unique a and B subgenomes. Plant J. 2020;103:32–52.

Jacobs G, Dechyeva D, Menzel G, Dombrowski C, Schmidt T. Molecular characterization of Vulmar1, a complete mariner transposon of sugar beet and diversity of mariner- and En/Spm-like sequences in the genus *Beta*. Genome. 2004;47:1192–1201.

Kubis S, Heslop-Harrison J, Schmidt T. A family of differentially amplified repetitive DNA sequences in the genus *Beta* reveals genetic variation in *Beta vulgaris* subspecies and cultivars. J Mol Evol. 1997;44:310–320.

Li N, Li X, Zhou J, Yu L, Li S, Zhang Y, et al. Genome-wide analysis of transposable elements and satellite DNAs in *Spinacia* species to shed light on their roles in sex chromosome evolution. Front Plant Sci. 2021;11:575462.

Maiwald S, Weber B, Seibt KM, Schmidt T, Heitkam T. The Cassandra retrotransposon landscape in sugar beet (*Beta vulgaris*) and related Amaranthaceae: recombination and re-shuffling lead to a high structural variability. Ann Bot. 2020;127:91–109.

Menzel G, Dechyeva D, Keller H, Lange C, Himmelbauer H, Schmidt T. Mobilization and evolutionary history of miniature inverted-repeat transposable elements (MITEs) in *Beta vulgaris* L. Chromosome Res. 2006;14:831–844.

Menzel G, Krebs C, Diez M, Holtgräwe D, Weisshaar B, Minoche AE, et al. Survey of sugar beet (*Beta vulgaris* L.) hAT transposons and MITE-like hATpin derivatives. Plant Mol Biol. 2012;78:393–405.

Novák P, Neumann P, Macas J. Global analysis of repetitive DNA from unassembled sequence reads using RepeatExplorer2. Nat Protoc. 2020;15:3745–3776.

Paesold S, Borchardt D, Schmidt T, Dechyeva D. A sugar beet (*Beta vulgaris* L.) reference FISH karyotype for chromosome and chromosome-arm identification, integration of genetic linkage groups and analysis of major repeat family distribution. Plant J. 2012;72:600–611.

Schmidt N, Seibt KM, Weber B, Schwarzacher T, Schmidt T, Heitkam T. Broken, silent, and in hiding: tamed endogenous pararetroviruses escape elimination from the genome of sugar beet (*Beta vulgaris*). Ann Bot. 2021;128:281–299.

Schmidt N, Sielemann K, Breitenbach S, Fuchs J, Pucker B, Weisshaar B, et al. Repeat turnover meets stable chromosomes: repetitive DNA sequences mark speciation and gene pool boundaries in sugar beet and wild beets. Plant J. 2023; <https://doi.org/10.1111/tpj.16599>.

Schmidt T, Jung C, Metzlaff M. Distribution and evolution of two satellite DNAs in the genus *Beta*. Theoret Appl Genetics. 1991;82:793–799.

Schmidt T, Heslop-Harrison JS. Variability and evolution of highly repeated DNA sequences in the genus *Beta*. Genome. 1993;36:1074–1079.

Schmidt T, Schwarzacher T, Heslop-Harrison JS. Physical mapping of rRNA genes by fluorescent *in-situ* hybridization and structural analysis of 5S rRNA genes and intergenic spacer sequences in sugar beet (*Beta vulgaris*). Theoret Appl Genetics. 1994;88:629–636.

Schmidt T, Heslop-Harrison JS. High-resolution mapping of repetitive DNA by *in situ* hybridization: molecular and chromosomal features of prominent dispersed and discretely localized DNA families from the wild beet species *Beta procumbens*. Plant Mol Biol. 1996;30:1099–1113.

Schwichtenberg K, Wenke T, Zakrzewski F, Seibt KM, Minoche A, Dohm JC, et al. Diversification, evolution and methylation of short interspersed nuclear element families in sugar beet and related Amaranthaceae species. Plant J. 2016;85:229–244.

Weber B, Schmidt T. Nested Ty3-*gypsy* retrotransposons of a single *Beta procumbens* centromere contain a putative chromodomain. Chromosome Res. 2009;17:379–396.

Weber B, Wenke T, Frömmel U, Schmidt T, Heitkam T. The Ty1-*copia* families SALIRE and Cotzilla populating the *Beta vulgaris* genome show remarkable differences in abundance, chromosomal distribution, and age. Chromosome Res. 2010;18:247–263.

Weber B, Heitkam T, Holtgräwe D, Weisshaar B, Minoche AE, Dohm JC, et al. Highly diverse chromoviruses of *Beta vulgaris* are classified by chromodomains and chromosomal integration. Mobile DNA. 2013;4:1–16.

Wollrab C, Heitkam T, Holtgräwe D, Weisshaar B, Minoche AE, Dohm JC, et al. Evolutionary reshuffling in the Errantivirus lineage Elbe within the *Beta vulgaris* genome. Plant J. 2012;72:636–651.

Zakrzewski F, Wenke T, Holtgräwe D, Weisshaar B, Schmidt T. Analysis of a c0t-1 library enables the targeted identification of minisatellite and satellite families in *Beta vulgaris*. BMC Plant Biol. 2010;10:1–14.

Zakrzewski F, Weisshaar B, Fuchs J, Bannack E, Minoche AE, Dohm JC, et al. Epigenetic profiling of heterochromatic satellite DNA. Chromosoma. 2011;120:409–422.

Zakrzewski F, Weber B, Schmidt T. A molecular cytogenetic analysis of the structure, evolution, and epigenetic modifications of major DNA sequences in centromeres of *Beta* species. In: Jiang J, Birchler JA, editors. Plant centromere biology. Oxford: John Wiley & Sons. 2013. p. 39–55.

Zakrzewski F, Schubert V, Viehoever P, Minoche AE, Dohm JC, Himmelbauer H, et al. The CHH motif in sugar beet satellite DNA: a modulator for cytosine methylation. Plant J. 2014;78:937–950.
